# An outer membrane vesicle specific lipoprotein promotes *Porphyromonas gingivalis* aggregation on red blood cells

**DOI:** 10.1016/j.crmicr.2024.100249

**Published:** 2024-06-08

**Authors:** Christina M. Rothenberger, Manda Yu, Hey-Min Kim, Yee-Wai Cheung, Yi-Wei Chang, Mary Ellen Davey

**Affiliations:** a Department of Microbiology, ADA Forsyth Institute, Cambridge, MA 02142, USA; b Department of Oral Microbiology, University of Florida College of Dentistry, University of Florida, Gainesville, FL, USA; c Department of Biochemistry and Biophysics, Perelman School of Medicine, University of Pennsylvania, Philadelphia, PA, USA; d Institute of Structural Biology, Perelman School of Medicine, University of Pennsylvania, Philadelphia, PA, USA; e Institute of Plant and Microbial Biology, Academia Sinica, Taipei, Taiwan

**Keywords:** OMVs, Fimbriae, Cell aggregation, Lipoprotein, Red blood cell, *P. gingivalis*

## Abstract

*Porphyromonas gingivalis* uses a variety of mechanisms to actively interact with and promote the hydrolysis of red blood cells (RBCs) to obtain iron in the form of heme. In this study, we investigated the function of lipoprotein PG1881 which was previously shown to be up-regulated during subsurface growth and selectively enriched on outer membrane vesicles (OMVs). Our results show that wildtype strain W83 formed large aggregates encompassing RBCs whereas the PG1881 deletion mutant remained predominately as individual cells. Using a PG1881 antibody, immunofluorescence revealed that the wildtype strain's aggregation to RBCs involves an extracellular matrix enriched with PG1881. Our findings discover that RBCs elicit cell aggregation and matrix formation by *P. gingivalis* and that this process is promoted by an OMV-specific lipoprotein. We propose this strategy is advantageous for nutrient acquisition as well as dissemination from the oral cavity and survival of this periodontal pathogen.

## Highlights

• PG1881 is an OMV-targeted fimbriae-like lipoprotein.
• PG1881 promotes *P. gingivalis* aggregation on red blood cells.
• PG1881 is potentially involved in membrane remodeling.
• Post-translational modifications are required for PG1881 subcellular localization.

## Graphical abstract

**Figure:**
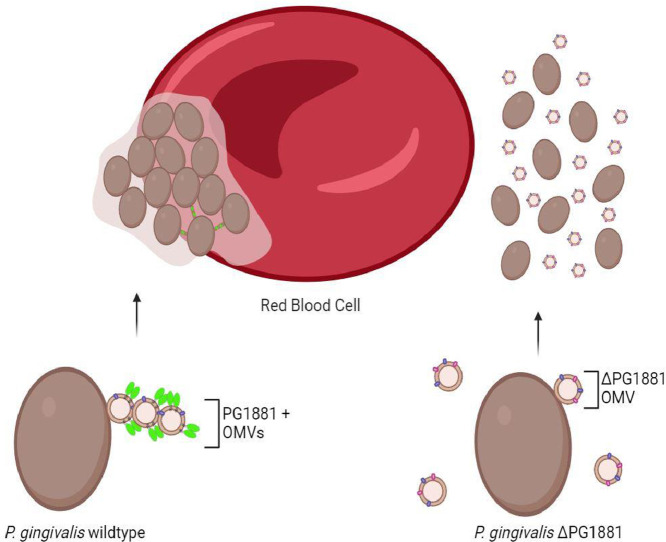

## 1 Introduction

Bacteria produce outer membrane vesicles (OMVs) to modify their environments and this behavior mediates a variety of fundamental processes, including nutrient acquisition, interspecies interactions, interkingdom signaling, and biofilm development (Mashburn-Warren and Whiteley, 2006; Orench-Rivera and Kuehn, 2016; Toyofuku et al., 2019; Stanton, 2021, 2023). Studies have shown that the periodontal pathogen *Porphyromonas gingivalis* is prolific in the production of OMVs and it produces copious amounts of OMVs under heme limiting conditions (Smalley et al., 1991), or when grown as a colony biofilm on the surface of blood agar (Kim and Davey, 2020). Intriguingly, when *P. gingivalis* is grown sandwiched between a hard surface and nutrient limited soft agar (subsurface growth), it also produces high levels of OMVs, specifically long interconnected chains of OMVs (Vermilyea et al., 2021). Most of the *P. gingivalis* OMV cargo proteins, including the well-characterized gingipains, are transported to the cell surface via a type IX secretion system (T9SS) (O-Brien-Simpson et al., 2003; Veith et al., 2014; Gui et al., 2016, 2017). This results in the selective release of a variety of virulence determinants that serve key functions as proteases, protein modifying enzymes, hemagglutinins, and adhesins (Lasica et al., 2017). Importantly, this particular species of *Porphyromonas* inhabits the subgingival crevice where it persists in intimate contact with the gingival tissues and encounters an array of host cells, including red blood cells (RBCs) (Hasiuk et al., 2016). In addition, it can translocate from a periodontal lesion into the circulation, where it also encounters RBCs (Forner et al., 2006). During subsurface growth, which we use to simulate subgingival growth conditions *in vitro, P. gingivalis* actively interacts with and promotes the hydrolysis and clearing of RBCs (Moradali et al., 2019). Key substrates that are known to influence *P. gingivalis* physiology are found in RBCs, including polyamines, oxoglutarate, pyruvate, lactate, and hemoglobin (Chen et al., 2004; Sakanaka et al., 2015; Moradali and Davey, 2021, 2022; Biagiotti et al., 2023). Moreover, *P. gingivalis* is atypical in its metabolism. It does not obtain energy from carbohydrates, instead it relies on proteins (Moradali and Davey, 2021). Since this anaerobic bacterium obtains iron from heme (Lewis et al., 2006), the hemoglobin in RBCs is thought to provide both peptides and iron and be a critical substrate for survival (Fujimura et al., 1995; Okamoto et al., 1998; Dashper et al., 2004; Simpson et al., 2004).

In general, bacteria form aggregates and surround themselves with an extracellular matrix to support their survival. Here, we show that a lipoprotein (PG1881) that was previously shown to be up-regulated during subsurface growth (Moradali et al., 2019) and carried on OMVs (Veith et al., 2014; Rocha et al., 2021) is indeed selectively enriched on OMVs and acts to promote cell-to-cell aggregation*.* Specifically, this lipoprotein promotes *P. gingivalis* aggregation around RBCs. Lastly, PG1881 has high structural similarity to the primary fimbriae subunits (FimA and Mfa1) produced by *P. gingivalis*, which are both important cell surface structures that promote colonization and aggregation (Xu et al., 2016; Hasegawa and Nagano, 2021). Our findings indicate that PG1881 is a critical component of the extracellular OMV matrix that mediates cell-to-cell aggregation during subsurface growth, specifically promoting the binding of OMVs to the surface of RBCs.

## 2 Materials and methods

### 2.1 Bacterial strains and culture conditions

Bacterial strains and plasmids used in this study are shown in Table 1. *P. gingivalis* strain W83 and derivatives were grown on Trypticase Soy Agar plates supplemented with 5 µg ml^−1^ hemin, 1 µg ml^−1^ menadione, and 5 % defibrinated sheep blood (Northeast Laboratory Services) (BAPHK) at 37 °C in an anaerobic chamber (Coy Lab Products) with an atmosphere containing 5 % hydrogen, 10 % carbon dioxide, and 85 % nitrogen. Planktonic cultures of *P. gingivalis* were grown in Trypticase Soy Broth or Brain Heart Infusion Broth (Becton, Dickinson and Company) supplemented with 5 µg ml^−1^ hemin and 1 µg ml^−1^ menadione (TSBHK and BHIHK, respectively). *P. gingivalis* deletion mutants were maintained by supplementing BAPHK with 10 µg ml^−1^ erythromycin (Erm). *P. gingivalis* strains harboring pT-COW plasmids were maintained by supplementing BAPHK with 1 µg ml^−1^ tetracycline (Tet). *E. coli* strains were grown in Luria Broth (LB) (Becton, Dickinson, and Company) or on LB agar plates at 37 °C. *E. coli* plasmid strains were maintained by supplementing the media with 100 µg ml^−1^ ampicillin (Amp) or 50 µg ml^−1^ kanamycin (Kan).

**Table 1:** List of bacterial strains and plasmids used in this study.

Strain	Description	Source or Reference
***P. gingivalis***		
W83	Wild Type	Davey lab strain collection Originally from C. Mouton, Laval University, Quebec City, Canada
W83ΔPG1881	PG1881::Erm^r^ in strain W83	This study
W83ΔPG1881 pT-C1881	Complemented strain	This study
W83ΔPG1881 pT-C1881 C21A	Site-directed mutagenesis strain on amino acid 21 of PG1881 from cysteine to alanine	This study
W83ΔPG1881 pT-C1881 R54A_K55A	Site-directed mutagenesis strain on amino acid 54 and 55 of PG1881 from arginine and lysine respectively to alanine	This study
W83ΔPG1881 pT-C1881 S372A	Site-directed mutagenesis strain on amino acid 372 of PG1881 from serine to alanine	This study
W83ΔPG1345-PG1346	PG1345-PG1346::Erm^r^ in strain W83	This study; (Veith et al., 2022)
***E. coli***		
NEB Dh5α	Cloning strain	New England Biolabs
*E. cloni* 10G	Cloning strain	Lucigen
S17–1	Conjugation strain	Davey lab strain collection
**Plasmids**		
pT-C1881	Fusion of constitutive groES promoter to PG1881 gene in pT-COW, Amp^r^ Tet^r^	This study
pT-C1881 C21A	Amp^r^ Tet^r^	This study
pT-C1881 R54A_K55A	Amp^r^ Tet^r^	This study
pT-C1881 S372A	Amp^r^ Tet^r^	This study
pRham N—His SUMO	Kan^r^	Lucigen
pRham N—His SUMO-PG1881	Kan^r^	This study

### 2.2 Stabbing to the subsurface of soft agar plates

Subsurface grown *P. gingivalis* cells were prepared as previously described (Moradali et al., 2019; Kim et al., 2022) with some modifications. Briefly, *P. gingivalis* cells from a 4-day BAPHK plate were inoculated in TSBHK and grown for 24 hrs. The culture was then sub-cultured at a 1:10 dilution into pre-reduced TSBHK and grown overnight to an OD_600_ of 1.0, followed by delivering 1 μl of culture to the plastic surface below the soft agar. Subsurface grown cells were observed 72 hrs after anaerobic incubation to obtain the subsurface growth area of about 1.5 cm in diameter. In this study, the soft agar plates did not contain hemin and had 5 % sheep's blood. Our rationale was this would create growth conditions where *P. gingivalis* would have to acquire its iron from the RBCs. In experiments comparing subsurface growth in the absence of RBCs, soft agar media contained Todd Hewitt Broth (Becton, Dickinson, and Company), 5 µg ml^−1^ hemin, and 1 µg ml^−1^ menadione. At least three replicates were used for each set of samples for the microscopy experiments described below.

### 2.3 Construction of *P. gingivalis* strain W83 gene deletion mutants, complementation, and site-directed mutagenesis

All primers used in this study are listed in Table 2. Deletion and replacement of the entire coding region of the gene encoding PG1881 with *ermF* was achieved by generating an allelic replacement cassette with the NEBuilder HiFi DNA assembly cloning kit (New England BioLabs) using the instructions provided by the manufacturer, as previously described (Moye et al., 2016; Ghods et al., 2023). Briefly, primers were designed to generate upstream and downstream products flanking PG1881 as well as a promoter-less erythromycin resistance gene (*ermF*) from plasmid pVA2198 (Fletcher et al., 1995). Overlapping genetic fragments were created using PCR and plasmid pUC19 as a vector. The fragments were attached by NEBuilder HiFi Assembly, and the resulting recombinant plasmid was transformed into *E. coli* strain DH5α and used for sequencing confirmation by Genewiz, Inc from Azenta Life Sciences (Waltham, MA, USA). The sequence-confirmed plasmid was isolated and used to amplify a linear DNA fragment for transformation by electroporation into competent *P. gingivalis* W83 cells. Transformants were selected on BAPHK agar containing 10 µg ml^−1^ Erm and the genome sequence of W83ΔPG1881 clones were confirmed.

**Table 2:** Primers used in this study.

Primer Name	Sequence (5′−3′)	Use
PUC19 F	AAGCTTGGCGTAATCATGG	Vector backbone for PG1881 deletion cassette
PUC19 R	GAATTCACTGGCCGTCGTTTTAC	Vector backbone for PG1881 deletion cassette
1 kb upstream PG1881 F	TTGTAAAACGACGGCCAGTGAATTCTTGGAGTGGTACAATAAC	Upstream fragment of PG1881 deletion cassette
1 kb upstream PG1881 R	TCTTTTTTGTCATTGGTGTTAAAATTTGATATTAAAAAG	Upstream fragment of PG1881 deletion cassette
ΔPG1881 *ermF* F	ATTTTAACACCAATGACAAAAAAGAAATTGCC	*ermF* fragment of PG1881 deletion cassette
ΔPG1881 *ermF* R	CTCAGAGCCTTCCTACGAAGGATGAAATTTTTC	*ermF* fragment of PG1881 deletion cassette
500 bp downstream PG1881 F	TCATCCTTCGTAGGAAGGCTCTGAGAGACAAAC	Downstream fragment of PG1881 deletion cassette
500 bp downstream PG1881 R	ACGGCTCGGCTCGCTACGCTGTAGG	Downstream fragment of PG1881 deletion cassette
PG1881::*ermF* seq F	ACGCTGGGAGTAAAGACTAC	Sequencing of PG1881 deletion mutants
PG1881::*ermF* seq R	GCTGCGCTCTACGGCTCTTCGAG	Sequencing of PG1881 deletion mutants
pT-COW F	GGATTTGTAGAATGGAAGCCGGCGGCAC	Vector backbone for complementation plasmid pT-C1881
pT-COW R	TCTATCCAATTACACGGTGCCTGACTGC	Vector backbone for complementation plasmid pT-C1881
*groES* F	GACGCTTATTTCGATATTGGATAGATGCCCTGC	*P. gingivalis groES* promoter for complementation plasmid pT-C1881
*groES* R	TCGTAAGCATTGTTGCTTGGTTTGTTATTG	*P. gingivalis groES* promoter for complementation plasmid pT-C1881
PG1881 F	CCAAGCAACAATGCTTACGAAACTAAAAACAC	PG1881 for complementation plasmid pT-C1881
PG1881 R	TCCGTTAGCGAGGTGCTACAAATCCACGTCCTG	PG1881 for complementation plasmid pT-C1881
pT-C1881 upstream C21A R	GATGATTGGA**TGC**AGAAAATCCAATACAAGCC	Site-directed mutagenesis on amino acid 21 of PG1881 from cysteine to alanine; TGC à GCA
pT-C1881 downstream C21A F	TGGATTTTCT**GCA**TCCAATCATCCGGTACTGAC	Site-directed mutagenesis on amino acid 21 of PG1881 from cysteine to alanine; TGC à GCA
pT-C1881 upstream R54_K55 R	GTGTACCACC**TGCAGC**GGAGAGGTGTAGGTTGG	Site-directed mutagenesis on amino acid 54 and 55 of PG1881 from arginine and lysine respectively to alanine; AGAAAGà GCTGCA
pT-C1881 downstream R54_K55 F	ACACCTCTCC**GCTGCA**GGTGGTACACATGATCCAC	Site-directed mutagenesis on amino acid 54 and 55 of PG1881 from arginine and lysine respectively to alanine; AGAAAG à GCTGCA
pT-C1881 upstream S372A R	CCTTCTTGAT**TGC**GTCGGCCAGAGCCAT	Site-directed mutagenesis on amino acid 372 of PG1881 from serine to alanine; AGCà GCA
pT-C1881 downstream S372A F	GGCCGAC**GCA**ATCAAGAAGGCGAAAGCTG	Site-directed mutagenesis on amino acid 372 of PG1881 from serine to alanine; AGC à GCA
pT-COW seq F	TAACCCCGGAGCTGCAAGC	Sequencing of PG1881 complemented strain and site-directed mutagenesis strains
pT-COW seq R	ACGCGATGGATATGTTCTG	Sequencing of PG1881 complemented strain and site-directed mutagenesis strains
pSUMO PG1881 F	CGCGAACAGATTGGAGGTTCCAATCATCCGGTACTGACG	Cloning of PG1881 on pRham N—His SUMO
pSUMO PG1881 R	GTGGCGGCCGCTCTATTACAA ATCCACGTCCTGCTCGTG	Cloning of PG1881 on pRham N—His SUMO
1 kb upstream PG1345-PG1346 F	AAACGACGGCCAGTGAATTCCTTGGGCAAACACGGTCATG	Upstream fragment of PG1345-PG1346 deletion cassette
1 kb upstream PG1345-PG1346 R	AAGCTATCGGTGCCTTTTTCTCTTATAACTACCCAAC	Upstream fragment of PG1345-PG1346 deletion cassette
ΔPG1345-PG1346 *ermF* with promoter F	GAAAAAGGCACCGATAGCTTCCGCTATTG	*ermF* fragment of PG1345-PG1346 deletion cassette
ΔPG1345-PG1346 *ermF* with promoter R	TTCGTACAAGTCATCTTGACAACCACCC	*ermF* fragment of PG1345-PG1346 deletion cassette
1 kb downstream PG1345-PG1346 F	GTCAAGATGACTTGTACGAAGAAGGAATAGGAAAAC	Downstream fragment of PG1345-PG1346 deletion cassette
1 kb downstream PG1345-PG1346 R	ACCATGATTACGCCAAGCTTCGCTTCACAGCAGTCGATG	Downstream fragment of PG1345-PG1346 deletion cassette
PG1345-PG1346::*ermF* seq F	TCCATGATGCTGTAGCAGAC	Sequencing of PG1345-PG1346 deletion mutants
PG1345-PG1346::*ermF* seq R	GCTTGGGCGAATTTCTTGC	Sequencing of PG1345-PG1346 deletion mutants

Complementation of W83ΔPG1881 was achieved by inserting a functional copy of PG1881 under the control of the low level, constitutive *P. gingivalis groES* promoter into the plasmid pT-COW to produce pT-C1881 (Gardner et al., 1996; Vermilyea et al., 2019). The pT-C1881 plasmid was transformed into *E. coli* S17–1 by heat shock (Simon et al., 1983). Colonies were selected on LB agar plates containing 100 μg ml^−1^Amp. The transformant was mated with W83ΔPG1881 and then selected on BAPHK containing 50 μg ml^−1^ gentamicin (Gent) and 1 μg ml^−1^ Tet to yield W83ΔPG1881 pT-C1881.

To create a complemented PG1881 [C21A] strain, the appropriate mutations were inserted into primers: pT-C1881 upstream C21 R and pT-C1881 downstream C21 F. For complemented PG1881 [R54A_K55A] strain, the appropriate mutations were inserted into primers pT-C1881 upstream R54_K55 R and pT-C1881 downstream R54_K55 F. For complemented PG1881 [S372A] strain, the appropriate mutations were inserted into primers: pT-C1881 upstream S372 R and pT-C1881 downstream S372 F. All complemented PG1881 site-directed mutagenesis strains used pT-C1881 as the template. Plasmids yielding PG1881[C21A], PG1881[R54A_K55A], or PG1881 [S372A] were transformed into *E. coli* S17–1 by heat shock. Colonies were selected on LB agar plates containing 100 μg ml^−1^ Amp. The transformant was mated with W83ΔPG1881 and then selected on blood agar plates containing 50 μg ml^−1^ Gent and 1 μg ml^−1^ Tet to yield W83ΔPG1881 pT-C1881 [C21A], W83ΔPG1881 pT-C1881 [R54A_K55A], or W83ΔPG1881 pT-C1881 [S372A].

To investigate the influence of O-glycosylation on PG1881, a double knockout of PG1345 and PG1346, group 1 glycosyltransferases involved in the biosynthesis of the O-glycan (Veith et al., 2022), were generated in *P. gingivalis* strain W83. Construction of the double mutant was similar to generation the PG1881 deletion mutant described above with the exception that the *ermF* promoter region was included for the PG1345-PG1346 deletion cassette.

### 2.4 Protein expression and purification

PG1881, from amino acids S22-L480, was cloned into the *E. coli* expression vector pRham N—His SUMO (Lucigen). Primer design, cloning, induction of protein expression, affinity purification, and removal of the purification tag were performed according to the protocol provided by the manufacturer and as previously described (Kim et al., 2021). Purified recombinant PG1881 (rPG1881) protein samples were stored at −20 °C.

### 2.5 Colony OMV preparation

OMVs from agar plate biofilms were isolated as previously described with some modifications (Cooke et al., 2019). Briefly, *P. gingivalis* strains were grown anaerobically in BHIHK for 24 hrs at 37 °C followed by inoculating BHIHK blood agar plates with 1 ml of culture and pipetting off the excess. For strain W83ΔPG1881 pT-C1881 and site-directed mutagenesis strains PG1881 [C21A], PG1881 [R54A_K55A] and PG1881 [S372A], BHIHK liquid culture and blood agar plates were supplemented with 1 µg ml^−1^ Tet. The biofilms were then grown anaerobically for 48 hrs and harvested into 0.85 % saline with 5 mM TLCK inhibitor (Abcam) and 1X protease inhibitor cocktail (Thermo Fisher Scientific) using a cell scraper. OMVs were liberated from the biofilm by vortexing. Specifically, the removed biofilm was vortexed in 0.85 % saline for 5 min at 4 °C, then centrifuged for 20 min at 4200 × *g* at 4 °C to pellet the cells. Afterwards, the supernatant was retained, the pellet was resuspended in 0.85 % saline with 5 mM TLCK inhibitor and 1X protease inhibitor cocktail, and the process was repeated one additional time. The retained supernatants were then pooled together and centrifuged once more for 20 min at 4200 × *g* at 4 °C to pellet any remaining cells, followed by filtration though a 0.22-micron PES filter. From this step, the supernatants were ultracentrifuged at 119,000  ×  *g* for 2 hrs at 4 °C to pellet crude colony OMVs which were then resuspended in 1X PBS containing 5 mM TLCK inhibitor and 1X protease inhibitor cocktail. Protein concentration of crude colony OMVs were determined using the Pierce™ BCA Protein Assay Kit (Thermo Fisher Scientific).

### 2.6 Whole cell lysate preparation

*P. gingivalis* cells were collected from colony biofilm cells described in the colony OMV preparation. Specifically, 50 µl of cell pellet from each strain were resuspended in 250 µl of B-PER™ Complete Bacterial Protein Extraction Reagent (Thermo Fisher Scientific) with 1X protease inhibitor cocktail and incubated for 5 min at room temperature with gentle rocking. Afterwards, samples were centrifuged at 20,000 x g for 10 min. The supernatant collected was designated as whole cell lysate and protein concentration was determined using the Pierce™ BCA Protein Assay Kit.

### 2.7 Production of anti-PG1881 antiserum

Based on the predicted 3D structure of PG1881 from AlphaFold (Q7MTR3), the peptide sequence LSSGTNEAYGDNSPL in an exposed loop region was chosen for antibody production. Peptide conjugation to Keyhole Limpet Hemocyanin (KLH) was synthesized by Peptide 2.0 (Chantilly, VA, USA) subsequently used for rabbit injections by Lampire Biological Laboratories (Pipersville, PA, USA) to produce polyclonal PG1881 antiserum.

### 2.8 Immunoblots

Colony OMVs and whole cell lysate samples from colony biofilms as well as rPG1881 were mixed with 4X SDS Sample Buffer and denatured by heating at 100 °C for 5 min. Colony OMV samples (1 µg), whole cell lysates (10 μg), and rPG1881 (50 ng) were then electrophoresed on a 10 % polyacrylamide Bis-Tris gel. For western blot analysis, proteins were transferred to a PVDF membrane. The membrane was blocked overnight in 1X PBS containing 0.1 % Tween 20 (PBS-T) and 1X Pierce™ Clear Milk Blocking Buffer (Thermo Fisher Scientific) with rocking at 4 °C. Primary PG1881 antisera (Lampire; this study) was added 1:1000 and incubated with the membrane for 1 hr with rocking at room temperature. After washing the membrane three times in PBS-T for 5 min each, the membrane was incubated with horseradish peroxidase-conjugated anti-rabbit IgG antibody (Cell Signaling Technology Cat# 7074) at 1:3000 for 1 hr with rocking at room temperature. The membrane was washed three times in PBS-T for 5 min before detecting bands using SuperSignal^R^ West Pico Chemiluminescent Substrate (Thermo Fisher Scientific).

### 2.9 Microscopy

For quantification of *P. gingivalis* cells surrounding red blood cells, subsurface grown *P. gingivalis* cells were collected by removing the soft agar after 72 hrs and the cells remaining on the bottom of the plastic surface were collected and fixed with 500 µl of 1X PBS plus 2 % paraformaldehyde. 100 µl of sample was then centrifuged at 10,000 x g for 2 min to remove fixative solution and resuspended in 10 µl of 1X PBS. Subsequently, 1 µl of sample was spotted onto a microscope slide with a coverslip. Images were acquired with Zeiss LSM 880 confocal upright microscope equipped with a 32-channel GaAsP Airyscan detector for super-resolution capabilities. Transmitted light images were captured using the T-PMT detector. The number of red blood cells surrounded by more than 10 *P gingivalis* cells were counted over the total number of red blood cells in the field of view and was quantified as a percentage. Ten images were used for parent strain W83 and W83ΔPG1881 to obtain an average percentage (Supplementary Fig. 2).

To perform immunofluorescence, PG1881 antisera was first labeled with Alexa Fluor® 647 Antibody labeling kit (Molecular Probes, Thermo Fisher Scientific) using the instructions provided by the manufacturer. In brief, 100 µl of PG1881 antisera was added to the amine reactive Alexa Fluor® 647 dye and incubated for 1 hr at room temperature followed by purification using a size exclusion spin column. For sample preparation, subsurface grown *P. gingivalis* cells were collected as described for the quantification experiments. After removing the fixative solution, cells were resuspended in 1X PBS with 1X Pierce™ Clear Milk solution and blocked for 15 min with gentle rocking at room temperature. Alexa Fluor® 647 conjugated PG1881 antisera was added to the blocking solution at a dilution of 1:100 and incubated with the samples for 15 min, protected from light and with gentle rocking. Additionally, 1 µl of SYTO 9 (Molecular Probes, Thermo Fisher Scientific) from a 1.67 µM stock solution was added to the sample to detect DNA. In experiments using N—Hydroxysuccinimide-Fluorescein (NHS-F; Thermo Fisher Scientific), which is an ester-labeling reagent that binds with primary amino groups (-NH_2_), 1 µl from a 1 mg ml^−1^ stock solution was added to the sample after primary antibody labeling for 15 min, protected from light. Samples were then centrifuged at 7000 x g for 2 min to remove unbound primary antibody and SYTO 9/NHS-F. Samples were gently re-suspended in 1X PBS, centrifuged once more, and then in a final suspension of 10 µl of 1X PBS where 1 µl of sample was spotted onto a microscope slide with a coverslip. Images were acquired with the same microscope described above. The detection of PG1881 antisera conjugated to Alexa Fluor® 647 was performed using a 633 nm excitation laser. The detection of SYTO 9 or NHS-F was performed using a 488 nm excitation laser. Transmitted light images were captured using the T-PMT detector. All images were acquired using the 63X objective.

For staining extracellular matrix, subsurface cells were treated with FilmTracer SYPRO Ruby biofilm matrix stain (Molecular Probes, Thermo Fisher Scientific) by adding 200 µl of solution to the samples followed by a 30 min incubation in the dark. SYTO 9 was added as described above. Samples were then centrifuged at 7000 x g for 2 min to remove unbound SYPRO ruby stain and SYTO 9. Samples were gently re-suspended in 1X PBS, centrifuged once more, and then in a final suspension of 10 µl of 1X PBS where 1 µl of sample was spotted onto a microscope slide with a coverslip. Images were acquired with the same microscope described above. The detection of SYTO 9 and transmitted light images were with same settings as described above, and detection of SYPRO Ruby biofilm matrix stain was performed using a 488 nm excitation laser. All images were acquired using the 63X objective.

Transmission electron microscopy (TEM) was carried out in the Electron Microscopy Resource Lab at the Perelman School of Medicine, University of Pennsylvania. In brief, subsurface grown cells were harvested and fixed in PBS with 2 % paraformaldehyde and were deposited onto glow-discharged carbon-coated copper grid and incubated for 1 min. The grid was subsequently negatively stained with 1 % (w/v) uranyl acetate for 30 s and air-dried. Images were acquired using FEI Tecnai T12 transmission electron microscope at an accelerating voltage of 100 kV. Aliquots of the same samples were immunogold labeled in a manner similar as described above for immunofluorescence using anti-PG1881 as the primary antibodies and anti-Rabbit IgG (*H* + *L*) Alexa Fluor™ 488–10 nm colloidal gold secondary antibodies (Thermo Fisher Scientific Cat# A-31,566).

### 2.10 Mass spectrometry and analysis

The PG1881 band from W83 crude colony OMVs and rPG1881 as well as an empty gel piece from W83ΔPG1881 crude colony OMVs at the same molecular weight (negative control) were excised from a 12 % polyacrylamide SDS-PAGE gel stained with Coomassie G250 (BioRad). The samples were then used for in-gel tryptic digestion. Mass spectrometry and analysis were performed as previously described (Hendrickson et al., 2022).

### 2.11 Hemagglutination assay

The hemagglutination activity was carried out as described previously (Shoji et al.*,* 2014) with modifications. Overnight planktonic cultures of *P. gingivalis* strains grown in TSBHK medium or subsurface cells grown for 72 hrs were centrifuged, washed, and suspended in PBS at OD_600_=3. The bacterial suspensions were then diluted in a two-fold series with 1X PBS in a round-bottom microtiter plate. A 100-μl aliquot of each suspension was mixed with an equal volume of sheep red blood cells (Innovative Research, final concentration 2 % in PBS,) and incubated at room temperature for 3 hrs. Negative control is sheep red blood cells mixed with 1X PBS only.

## 3 Results & discussion

### 3.1 PG1881 promotes aggregation of *P. gingivalis* and attachment on RBCs

**Fig. 1:**
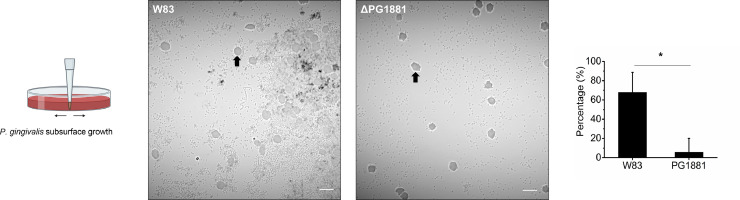
PG1881 promotes aggregation of *P. gingivalis* and attachment on red blood cells (RBCs). (A) Representative confocal microscopy images of parent strain W83 and PG1881 deletion mutant cells grown on the subsurface of soft blood agar plates at 72 hrs. Black arrows indicate RBCs. Scale bar represents 10 µm. The graphic on the left illustrates *P. gingivalis* subsurface growth conditions. The bar graph on the right represents quantification of the percentage of RBCs surrounded by more than ten *P. gingivalis* cells from confocal microscopy images. Data are representative of three independent experiments showing similar results and are presented as mean ± standard deviation (*n* = 10 images per strain). Data were analyzed using Student's two-tailed *t*-test with unequal variance. *p < 0.05.

When *P. gingivalis* is growing at the subsurface of soft agar, it is limited for nutrients. Our working model has been that it produces copious amounts of individual and long interconnected chains of OMVs, thereby spreading proteases and heme-binding proteins into the surroundings which promotes acquisition of essential nutrients (peptides and heme) (Gui et al., 2016; Moradali et al., 2019). In addition, PG1881 gene expression is detected at higher levels under these growth conditions (Moradali et al., 2019). To understand the role of PG1881, we compared the macroscopic phenotype between parent strain W83 and the W83ΔPG1881 mutant when grown at the subsurface as well as when grown on the surface of blood agar. Although no differences were observed when cells were grown on the surface of blood agar plates, we found that when grown at the subsurface, the parent strain W83 had a darker pigmentation than W83ΔPG1881 which is indicative of heme accumulation (Supplementary Fig. 1A). To further analyze this macroscopic phenotype, we microscopically examined the subsurface growing cells, and it was readily apparent that the parent strain W83 formed large aggregates encompassing RBCs whereas the PG1881 deletion mutant did not. The mutant cells remained as individual cells with few surface-attached microcolonies (Fig. 1). Quantification of confocal microscopy images determined that, on average, the percentage of RBCs surrounded by *P. gingivalis* aggregates is distinctly higher in parent strain W83 (Fig. 1, bar graph; Supplementary Fig. 2 used for quantification). Additionally, in a complemented strain where PG1881 is expressed from a plasmid under the control of a low-level constitutive promoter *groES*, aggregates formed even when RBCs were absent in the media (cPG1881, Supplementary Fig. 3), which supports the potential role of PG1881 in aggregate formation.

**Fig. 2:**
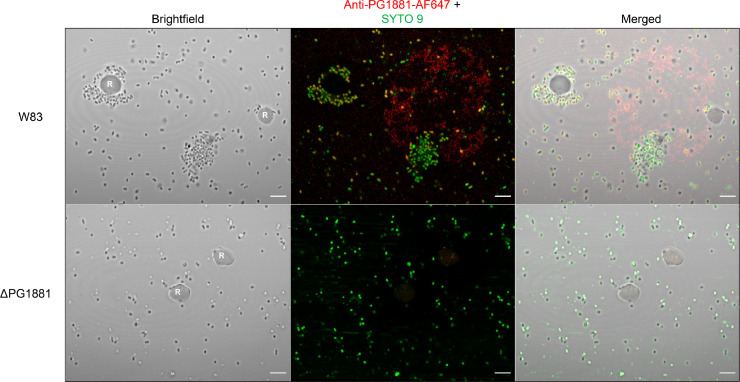
PG1881 plays a role in extracellular matrix development and aggregate formation around RBCs. Immunofluorescent staining of PG1881 (red fluorescence) on *P. gingivalis* cells during subsurface growth at 72 hrs. Top panels are parent strain W83 and bottom panels are the PG1881 deletion mutant. SYTO 9 was used to stain for DNA (green fluorescence). For all images, magnification is 63X with Zeiss Airyscan image processing. R indicates RBC. Scale bar represents 5 µm.

Furthermore, localization of PG1881 during subsurface growth was visualized using PG1881 antibody conjugated to Alexa Fluor® 647 for immunofluorescence. In strain W83, fluorescence signal for PG1881 was detected on cell aggregates near RBCs and was located extracellularly (Fig. 2, top panels), while there was negligible signal in the negative control (PG1881 deletion mutant; Fig. 2, bottom panels). Interestingly, PG1881 immunofluorescence signal was not only detected in the extracellular matrix of the bacterial cell aggregates but also as separate entities away from the bacterial cells on the surface of RBCs and this included what appeared to be OMV complexes (Supplementary Fig. 1B, top middle and right panels). This result indicates that PG1881-containing OMVs are released and attach to the surface of RBCs, and potentially prepare the surface for attachment of *P. gingivalis* cells. Lastly, hemagglutination assays revealed higher activity in parent strain W83 compared to the PG1881 deletion mutant, but only when grown at the subsurface (Supplementary Fig. 2C). Overall, the data show that PG1881 plays a key role in extracellular matrix development and aggregate formation around RBCs.

**Fig. 3:**
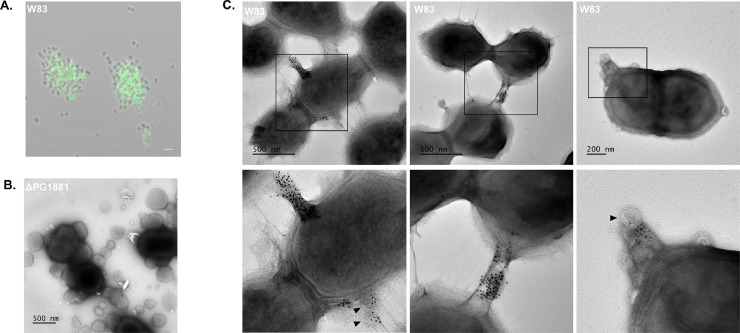
PG1881 may be required for remodeling of OMVs and outer membrane to form protrusions for cell-to-cell connections. (A) Confocal imaging of parent strain W83 using Alexa Fluor 488. Scale bar represents 2 µm. (B) TEM was performed to compare the subsurface growth between parent strain W83 and the PG1881 deletion mutant. Immunogold electron microscopy of W83ΔPG1881 show large vesicles extending from the membrane that were not observed in parent strain W83. (C) Immunogold electron microscopy to detect PG1881 on subsurface growth from parent strain W83. Black arrows (left and right panels) point to OMVs.

To further verify the subcellular location of PG1881 on the bacterial cells, immunogold electron microscopy was performed using PG1881 primary antibodies and secondary AF488-AuNP (10 nm) conjugated antibodies. As the secondary antibodies were dual-labeled, we first confirmed the immunostaining was successful and observed PG1881 signal mainly on cell aggregates (Fig. 3A). For the immunogold images, AuNPs were detected on OMVs (Fig. 3C, left and right panels, black arrows), but the majority of AuNPs accumulated on membrane protrusions that carried OMVs or bridged two *P. gingivalis* cells (Fig. 3C, left and middle panels). These images indicate that during subsurface nutrient limited growth, PG1881 protein is transported to select sites on the cell surface, and we propose that this lipoprotein functions to support OMVs in remodeling of the outer membrane to generate tube-like extensions. There has been evidence of diverse outer membrane tube extensions across different bacteria with various functions (Toyofuku et al., 2019; Kaplan et al., 2021), and our data support the involvement of extracellular structures as elements supporting metabolic processes associated with nutrient acquisition from RBCs, yet how these structures are formed, and their true function is not yet clear. Additionally, no AuNPs were observed in the PG1881 deletion mutant, and a subset of the cells produced large vesicles extending from the membrane (Fig. 3B). Whether PG1881 influences OMV membrane stability during subsurface growth needs further investigation.

### 3.2 RBCs elicit aggregate and matrix formation in *P. gingivalis*

It has previously been observed that *P. gingivalis* hydrolyzes RBCs under subsurface growth conditions (Moradali et al., 2019), and we posit this interaction mediates nutrient acquisition. We investigated the interaction between *P. gingivalis* and RBCs by comparing subsurface cells from strain W83 grown in the presence or absence of RBCs. The results show that under these growth conditions strain W83 forms aggregates that surround the RBCs (Supplementary Fig. 3, top left panel). In contrast, *P. gingivalis* mainly exists as an individual cell or in pairs when grown in the absence of RBCs (Supplementary Fig. 3, top right panel).

**Fig. 4:**
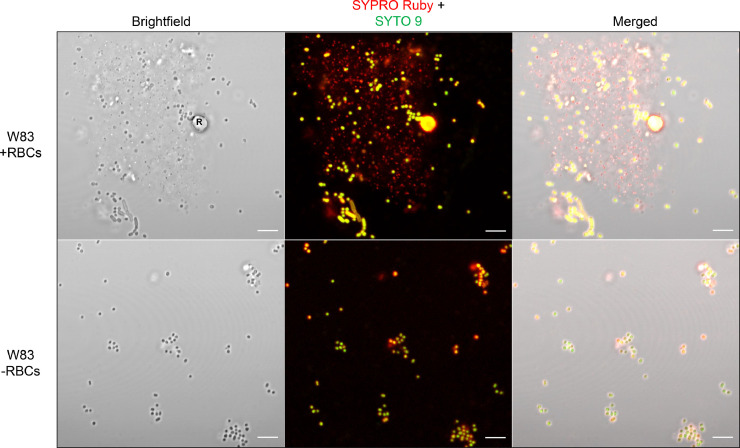
RBCs promote matrix formation by *P. gingivalis* strain W83. Fluorescent staining of protein (red fluorescence) and DNA (green fluorescence) on *P. gingivalis* during subsurface growth at 72 hrs. Top panels show *P. gingivalis* grown in the presence of RBCs and bottom panels are cells grown in the same medium without RBCs. For all images, magnification is 63X with Zeiss Airyscan image processing. R indicates RBC. Scale bar represents 5 µm.

Next, subsurface grown cells were labeled with SYTO 9 and SYPRO Ruby to stain DNA and protein respectively. In the presence of RBCs, *P. gingivalis* cells labeled with both SYTO 9 and SYPRO Ruby, and copious amounts of nanosized extracellular particles labeled with SYPRO Ruby were observed, suggesting an extracellular protein matrix containing OMVs was formed (Fig. 4, top panels). In contrast, when W83 was grown in the absence of RBCs, extracellular matrix formation was not detected (Fig. 4, bottom panels). Collectively, the data indicate that RBCs influence cell-to-cell interactions as well as matrix formation in *P. gingivalis* during subsurface growth.

### 3.3 Delivery of PG1881 to OMVs requires a cysteine residue known to be conserved in lipoproteins

**Fig. 5:**
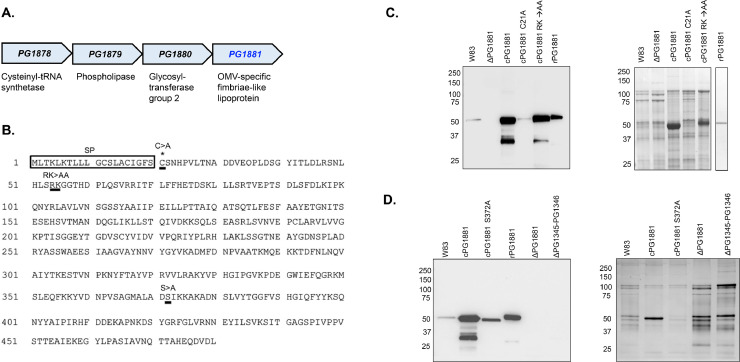
PG1881 operon, post-translational modifications, and localization. (A) Operon encoding PG1881, an OMV-specific fimbriae-like lipoprotein. (B) Amino acid sequence of PG1881. Black box indicates signal peptide sequence that is subsequently cleaved by signal peptidase. Underlined cysteine with a single * is the residue predicted to be lipidated and C > A indicates site-directed mutagenesis. Underlined arginine and lysine are predicted gingipain cleavage sites and the site-directed mutagenesis of these residues (RK>AA) are noted above. Underlined serine with two ** correspond to previously identified O-glycosylation site (Veith et al., 2022) and the site-directed mutagenesis of this residue (*S* > *A*) is noted above. (C and D, left images) Western blot analyses of crude colony OMVs using PG1881 antibody show that PG1881 is localized to OMVs, C21 is required for its exportation, R54/K55 are potential gingipain cleavage sites, and O-glycosylation affects PG1881 protein stability. On the right is the corresponding Coomassie stain gels.

PG1881 is annotated as a lipoprotein that is conserved across multiple wildtype strains (Nagano et al., 2017), and shown to specifically localize to OMVs (Veith et al., 2014; Xu et al., 2016; Rocha et al., 2021), but characterization of this lipoprotein is limited. Typically, in Gram-negative bacteria, lipoprotein transport includes a signal peptide sequence that allows translocation to the inner membrane by the secretory (Sec) complex, a conserved cysteine residue +1 from the signal peptide for lipidation, cleavage of the signal peptide by a signal peptidase, and transport to the outer membrane by the localization of lipoprotein (Lol) pathway (Nakayama et al., 2012; Wilson and Bernstein, 2016). An example of the importance of the conserved cysteine residue for lipoprotein transport in *P. gingivalis* was shown by conducting site-directed mutagenesis of Mfa2 which is the lipoprotein that acts as the anchor pilin for minor fimbriae Mfa1 (Xu et al., 2016). Specifically, fractionation analysis with western blotting showed that mutagenesis of the conserved cysteine to alanine resulted in no Mfa2 protein levels detected on the outer membrane fraction (Xu et al., 2016). Analysis of the PG1881 amino acid sequence with the bioinformatic tool SignalP 6.0 (Teufel et al., 2022) showed high probability of a signal peptidase cleavage site between amino acid S20 and C21 and notably lipidation at C21 (Fig. 5B).

To obtain a better understanding of PG1881 transport, site-directed mutagenesis was performed on the cysteine predicted to be lipidated by changing C21 to alanine (Fig. 5B). Western blot analyses using PG1881 antibody on crude colony OMV samples showed a band for PG1881 in the parent strain W83 and complemented strain while no signal was detected in the PG1881 deletion mutant strain (Fig. 5C). The stronger band intensity seen in the PG1881 complemented strain is attributed to the plasmid transcribing PG1881 under the constitutive *groES* promoter. In contrast, the PG1881 complemented strain with site directed mutagenesis on C21 exhibited reduced PG1881 protein levels from crude colony OMVs compared to the complemented strain with no mutations (Fig. 5C). Additionally, whole cell lysates showed that PG1881 protein levels in parent strain W83 and complemented strain were low compared to the protein levels detected on crude colony OMVs (Supplementary Fig. 4A), resonating the previous findings that PG1881 is enriched on OMVs (Veith et al., 2014; Xu et al., 2016; Rocha et al., 2021). In essence, the data indicates that amino acid C21 is critical for PG1881 transport to the outer membrane and subsequent loading onto OMVs, but the exact modification requires further investigation.

Although lipoprotein biosynthesis is not fully elucidated for *P. gingivalis*, a previous study determined the lipid modifications of *P. gingivalis* lipoprotein PG1828; more specifically, the authors discovered it was lipidated with two palmitoyl chains and one pentadecenoyl chain at the conserved cysteine residue (Hashimoto et al., 2004; Makimura et al., 2006). The addition of palmitoyl chains is particularly interesting because *P. gingivalis* synthesizes sphingolipids which are known to generate microdomains in the cell-membrane that support proteins that are palmitoylated (Nichols, 1998; Nichols et al., 2004; Moye et al., 2016). Moreover, it has been determined that PG1881 is absent in OMVs that lack sphingolipids (Rocha et al., 2021). Hence, our working model is that PG1881 is palmitoylated in a similar manner as PG1828 on the conserved cysteine residue which allows for association with sphingolipid microdomains once transported to the outer membrane. Experiments to determine the link between sphingolipids and the localization of PG1881 are on-going.

### 3.4 Post-translational modifications of PG1881 include proteolytic processing and O-glycosylation

Another potential modification of PG1881 is proteolytic processing by the gingipains (Xu et al., 2016; Nagano et al., 2017). Fimbrial subunits FimA and Mfa1, which have structural homology to PG1881, are exported to the outer membrane as lipoprotein precursors and their polymerization is protease-mediated, mainly by arginine gingipains (Shoji et al., 2004; Xu et al., 2016; Lee et al., 2018; Shibata et al., 2020; Hasegawa and Nagano, 2021). Previous studies examining *P. gingivalis* FimA and Mfa1 polymerization with arginine gingipain-null mutants or arginine and lysine gingipain-null mutants showed that the monomer was at a higher molecular weight than the monomer from the wildtype strain for both proteins and thereby were described as precursor forms (Shoji et al., 2004; Xu et al., 2016; Lee et al., 2018). Similarly, we observed in our western blots that the PG1881 band from *P. gingivalis* crude colony OMVs had a lower molecular weight than recombinant PG1881 (rPG1881) which is 51 kDa, suggesting proteolytic cleavage (Fig. 5C). Previous N-terminal sequencing of PG1881 from bacterial cell lysates showed that the first peptide detected started at amino acid G56 (Nagano et al., 2017). Therefore, we performed site-directed mutagenesis on R54 and K55 to alanine (Fig. 5B). Western blot analysis as well as Coomassie staining of crude colony OMVs showed that the complemented strain with R54A and K55A mutations had a slightly higher PG1881 band size compared to the complemented strain with no mutations (Fig. 5C) indicating that these amino acids are potential gingipain cleavage sites. Additional analysis was performed by excising the PG1881 band from crude colony OMVs in *P. gingivalis* for trypsin digestion followed by mass spectrometry. Our analysis shows that the first peptide detected begins at R67 (Supplementary Fig. 5), yet it is important to note that this result could be misleading since the trypsin digestion has the potential to cleave at the same site as the trypsin-like gingipains. Nonetheless, our data suggest PG1881 is proteolytically processed, however the exact cleavage site still needs to be determined by using non-arginine cutting proteinases for mass spectrometry analysis.

O-glycosylation, which is the addition of sugars to proteins at S/T residues, is a highly prevalent post-translational modification in gut *Bacteroides* species and was recently confirmed to be present in *P. gingivalis* (Fletcher et al., 2009, 2011; Veith et al., 2022). The motif for O-glycosylation D(S, T)(A,L,V,I,M,T) was previously found to be conserved across the Bacteroidetes phylum (Coyne et al., 2013) and the conserved motif DSI is detected in PG1881 (Veith et al., 2022). During our mass spectrometry analysis comparing rPG1881 to PG1881 from *P. gingivalis* crude colony OMVs, we noticed that the non-modified peptides (KKYVDNPVSAGMALADSIK/ YVDNPVSAGMALADSIK) covering the reported O-glycosylated serine (Veith et al., 2022) were only detected in rPG1881 but were absent in the *P. gingivalis* produced PG1881 (Supplementary Fig. 5). This finding supports previous studies reporting that this region of PG1881 is modified by O-glycosylation at a serine residue (Veith et al., 2022). To further investigate the significance of O-glycosylation on PG1881, the predicted O-glycosylated serine was converted to alanine in a complemented strain (Fig. 5B). Western blot analyses on crude colony OMVs show that the PG1881 band in the complemented strain with the mutated O-glycosylated serine was at a lower molecular weight than the band from the complemented strain with no mutations (Fig. 5D), indicating that the serine residue could be O-glycosylated. Similar results were previously observed in western blot analyses of *B. fragilis* glycoproteins with site-directed mutagenesis on the predicted O-glycosylated serine or threonine to alanine (Fletcher et al., 2011). Our results also suggest that loss of the O-glycan through site-directed mutagenesis impacted PG1881 transport to OMVs since the band intensity of the complemented strain with the mutated O-glycosylated serine was less than the complemented strain with no mutations, and there were higher PG1881 protein levels detected in the whole cell lysate in the serine mutant versus the complemented strain (Fig. 5D; Supplementary Fig. 4B). Lastly, we examined the glycosyltransferases previously identified to be involved in the biosynthesis of the O-glycan in *P. gingivalis* (Veith et al., 2022), PG1345 and PG1346, by generating a double deletion mutant in strain W83. Western blot analyses show that transport to OMVs and potentially synthesis of PG1881 was affected since there was no band detected on the crude colony OMVs nor the whole cell lysate (Fig. 5D; Supplementary Fig. 4B).

O-glycosylation is of interest because it is not a dominant sugar modification to outer membrane proteins in *P. gingivalis*, yet PG1881 as well as fimbrial proteins Mfa2 and Mfa5 were identified as targets (Veith et al., 2022). It has been suggested that this modification may provide protein stability and protection from proteases (Fletcher et al., 2009; Veith et al., 2022), so this potentially explains the reduced PG1881 protein levels observed in colony OMVs when the O-glycosylated serine was mutated or when the glycosyltransferases were absent. Interestingly, a putative group II glycosyltransferase (PG1880) is found in the same operon of PG1881 (Fig. 5A), suggesting glycosylation is likely essential for PG1881 function and deserves further investigation.

## 4 Conclusion

This study confirmed that the fimbriae-like lipoprotein PG1881 is highly specific to OMVs. The results also provide additional insights into the amino acid residues and post-translational modifications needed for transport of PG1881 to the outer membrane. Importantly, this study discovered that RBCs elicit aggregate and matrix formation of *P. gingivalis* and this process requires PG1881-containing OMVs. Specifically, immunogold labeling with anti-PG1881 antibodies followed by transmission electron microscopy imaging revealed that when *P. gingivalis* strain W83 is grown sandwiched between two surfaces under nutrient limited conditions, and in the presence of RBCs, PG1881-protein accumulates at select sites on the membrane of *P. gingivalis* cells and these focal points are sites where membrane protrusions containing OMVs are generated. We are proposing that this lipoprotein functions to support formation of a membrane protrusion that allows extension of the OMV from the cell surface out into the environment and sometimes these extensions form connections to other *P. gingivalis* cells within the aggregate. Thus, PG1881 appears to play a critical role in cell-to-cell connections during aggregate formation and colonization of red blood cells. In addition, while studies are showing that periodontal bacteria are detected in the bloodstream (Perez-Chaparro et al., 2008) and at distant parts of the body (Giordano-Kelhoffer et al., 2022; Peng et al., 2022) the process by which these bacteria traffic to distal locations and survive is not understood. Certain strains of *P. gingivalis*, including strain W83 have been detected at distant sites far from its natural habitat in the subgingival crevice (Gomez et al., 2020; de Jongh et al., 2023). The concept that RBCs can serve as a transport vehicle of *P. gingivalis* aggregates, allowing systemic spread via the vascular system and protection from immune cells has been proposed as one potential mechanism that supports dissemination (Belstrom et al., 2011; Hajishengallis, 2015; Damgaard et al., 2017) and the results presented here support this possibility. In conclusion, the concept that RBCs can promote aggregate (and matrix) formation by *P. gingivalis*, a behavior that is known to protect bacteria from harsh environments and promote survival is a significant step forward in our understanding of the basic physiology of *P. gingivalis* with implications into its link to systemic disease.

## CRediT authorship contribution statement

**Christina M. Rothenberger:** Project administration, Formal analysis, Writing – original draft, Writing – review & editing. **Manda Yu:** Formal analysis, Writing – original draft, Writing – review & editing. **Hey-Min Kim:** Formal analysis, Writing – review & editing. **Yee-Wai Cheung:** Formal analysis, Writing – review & editing. **Yi-Wei Chang:** Writing – review & editing. **Mary Ellen Davey:** Project administration, Writing – original draft, Writing – review & editing.

## Declaration of competing interest

The authors declare that they have no known competing financial interests or personal relationships that could have appeared to influence the work reported in this paper.

## Data Availability

No data was used for the research described in the article.

## Appendix Supplementary materials

Image, application 1

## References

[bib0001] Belstrom D., Holmstrup P., Damgaard C., Borch T.S., Skjodt M.O., Bendtzen K., Nielsen C.H. (2011). The atherogenic bacterium porphyromonas gingivalis evades circulating phagocytes by adhering to erythrocytes. Infect. Immun..

[bib0002] Biagiotti S., Pirla E., Magnani M. (2023). Drug transport by red blood cells. Front. Physiol..

[bib0003] Chen T., Hosogi Y., Nishikawa K., Abbey K., Fleischmann R.D., Walling J., Duncan M.J. (2004). Comparative whole-genome analysis of virulent and avirulent strains of porphyromonas gingivalis. J. Bacteriol..

[bib0004] Cooke A.C., Nello A.V., Ernst R.K., Schertzer J.W. (2019). Analysis of pseudomonas aeruginosa biofilm membrane vesicles supports multiple mechanisms of biogenesis. PLoS One.

[bib0005] Coyne M.J., Fletcher C.M., Chatzidaki-Livanis M., Posch G., Schaffer C., Comstock L.E. (2013). Phylum-wide general protein o-glycosylation system of the bacteroidetes. Mol. Microbiol..

[bib0006] Damgaard C., Kantarci A., Holmstrup P., Hasturk H., Nielsen C.H., Van Dyke T.E. (2017). Porphyromonas gingivalis-induced production of reactive oxygen species, tumor necrosis factor-alpha, interleukin-6, cxcl8 and ccl2 by neutrophils from localized aggressive periodontitis and healthy donors: modulating actions of red blood cells and resolvin e1. J. Periodontal. Res..

[bib0007] Dashper S.G., Cross K.J., Slakeski N., Lissel P., Aulakh P., Moore C., Reynolds E.C. (2004). Hemoglobin hydrolysis and heme acquisition by porphyromonas gingivalis. Oral Microbiol. Immunol..

[bib0008] de Jongh C.A., de Vries T.J., Bikker F.J., Gibbs S., Krom B.P. (2023). Mechanisms of porphyromonas gingivalis to translocate over the oral mucosa and other tissue barriers. J. Oral Microbiol..

[bib0009] Fletcher C.M., Coyne M.J., Comstock L.E. (2011). Theoretical and experimental characterization of the scope of protein o-glycosylation in bacteroides fragilis. J. Biol. Chem..

[bib0010] Fletcher C.M., Coyne M.J., Villa O.F., Chatzidaki-Livanis M., Comstock L.E. (2009). A general o-glycosylation system important to the physiology of a major human intestinal symbiont. Cell.

[bib0011] Fletcher H.M., Schenkein H.A., Morgan R.M., Bailey K.A., Berry C.R., Macrina F.L. (1995). Virulence of a porphyromonas gingivalis w83 mutant defective in the prth gene. Infect. Immun..

[bib0012] Forner L., Larsen T., Kilian M., Holmstrup P. (2006). Incidence of bacteremia after chewing, tooth brushing and scaling in individuals with periodontal inflammation. J. Clin. Periodontol..

[bib0013] Fujimura S., Shibata Y., Hirai K., Nakamura T. (1995). Some binding properties of the envelope of porphyromonas gingivalis to hemoglobin. FEMS Immunol. Med. Microbiol..

[bib0014] Gardner R.G., Russell J.B., Wilson D.B., Wang G.R., Shoemaker N.B. (1996). Use of a modified bacteroides-prevotella shuttle vector to transfer a reconstructed beta-1,4-d-endoglucanase gene into bacteroides uniformis and prevotella ruminicola b(1)4. Appl. Environ. Microbiol..

[bib0015] Ghods S., Moradali M.F., Duryea D., Walker A.R., Davey M.E. (2023). Growth of porphyromonas gingivalis on human serum albumin triggers programmed cell death. J. Oral Microbiol..

[bib0016] Giordano-Kelhoffer B., Lorca C., March Llanes J., Rabano A., Del Ser T., Serra A., Gallart-Palau X. (2022). Oral microbiota, its equilibrium and implications in the pathophysiology of human diseases: a systematic review. Biomedicines.

[bib0017] Gomez L.A., De Avila J., Castillo D.M., Montenegro D.A., Trujillo T.G., Suarez L.J., Lafaurie G.I. (2020). Porphyromonas gingivalis placental atopobiosis and inflammatory responses in women with adverse pregnancy outcomes. Front. Microbiol..

[bib0018] Gui M.J., Dashper S.G., Slakeski N., Chen Y.Y., Reynolds E.C. (2016). Spheres of influence: porphyromonas gingivalis outer membrane vesicles. Mol. Oral Microbiol..

[bib0019] Hajishengallis G. (2015). Periodontitis: from microbial immune subversion to systemic inflammation. Nat. Rev. Immunol..

[bib0020] Hasegawa Y., Nagano K. (2021). Porphyromonas gingivalis fima and mfa1 fimbriae: current insights on localization, function, biogenesis, and genotype. Jpn. Dent. Sci. Rev..

[bib0021] Hashimoto M., Asai Y., Ogawa T. (2004). Separation and structural analysis of lipoprotein in a lipopolysaccharide preparation from porphyromonas gingivalis. Int. Immunol..

[bib0022] Hasiuk P., Hasiuk N., Kindiy D., Ivanchyshyn V., Kalashnikov D., Zubchenko S. (2016). Characteristics of cellular composition of periodontal pockets. Interv. Med. Appl. Sci..

[bib0023] Hendrickson E.L., Bor B., Kerns K.A., Lamont E.I., Chang Y., Liu J., Cen L., Schulte F., Hardt M., Shi W., He X., McLean J.S. (2022). Transcriptome of epibiont saccharibacteria nanosynbacter lyticus strain tm7x during the establishment of symbiosis. J. Bacteriol..

[bib0024] Kaplan M., Chreifi G., Metskas L.A., Liedtke J., Wood C.R., Oikonomou C.M., Nicolas W.J., Subramanian P., Zacharoff L.A., Wang Y., Chang Y.W., Beeby M., Dobro M.J., Zhu Y., McBride M.J., Briegel A., Shaffer C.L., Jensen G.J. (2021). *In situ* imaging of bacterial outer membrane projections and associated protein complexes using electron cryo-tomography. Elife.

[bib0025] Kim H.M., Davey M.E. (2020). Synthesis of ppgpp impacts type ix secretion and biofilm matrix formation in porphyromonas gingivalis. NPJ Biofilms. Microbiomes.

[bib0026] Kim H.M., Ranjit D.K., Walker A.R., Getachew H., Progulske-Fox A., Davey M.E. (2021). A novel regulation of k-antigen capsule synthesis in porphyromonas gingivalis is driven by the response regulator pg0720-directed antisense rna. Front. Oral Health.

[bib0027] Kim H.M., Rothenberger C.M., Davey M.E. (2022). Cortisol promotes surface translocation of porphyromonas gingivalis. Pathogens.

[bib0028] Lasica A.M., Ksiazek M., Madej M., Potempa J. (2017). The type ix secretion system (t9ss): highlights and recent insights into its structure and function. Front. Cell Infect. Microbiol..

[bib0029] Lee J.Y., Miller D.P., Wu L., Casella C.R., Hasegawa Y., Lamont R.J. (2018). Maturation of the mfa1 fimbriae in the oral pathogen porphyromonas gingivalis. Front. Cell Infect. Microbiol..

[bib0030] Lewis J.P., Plata K., Yu F., Rosato A., Anaya C. (2006). Transcriptional organization, regulation and role of the porphyromonas gingivalis w83 hmu haemin-uptake locus. Microbiology.

[bib0031] Makimura Y., Asai Y., Taiji Y., Sugiyama A., Tamai R., Ogawa T. (2006). Correlation between chemical structure and biological activities of porphyromonas gingivalis synthetic lipopeptide derivatives. Clin. Exp. Immunol..

[bib0032] Mashburn-Warren L.M., Whiteley M. (2006). Special delivery: vesicle trafficking in prokaryotes. Mol. Microbiol..

[bib0033] Moradali M.F., Davey M.E. (2021). Metabolic plasticity enables lifestyle transitions of porphyromonas gingivalis. NPJ Biofilms. Microbiomes.

[bib0034] Moradali M.F., Ghods S., Angelini T.E., Davey M.E. (2019). Amino acids as wetting agents: surface translocation by porphyromonas gingivalis. ISMe J..

[bib0035] Moye Z.D., Valiuskyte K., Dewhirst F.E., Nichols F.C., Davey M.E. (2016). Synthesis of sphingolipids impacts survival of porphyromonas gingivalis and the presentation of surface polysaccharides. Front. Microbiol..

[bib0036] Nagano K., Hasegawa Y., Yoshida Y., Yoshimura F. (2017). Novel fimbrilin pgn_1808 in porphyromonas gingivalis. PLoS One.

[bib0037] Nakayama H., Kurokawa K., Lee B.L. (2012). Lipoproteins in bacteria: structures and biosynthetic pathways. FEBS J..

[bib0038] Nichols F.C. (1998). Novel ceramides recovered from porphyromonas gingivalis: relationship to adult periodontitis. J. Lipid Res..

[bib0039] Nichols F.C., Riep B., Mun J., Morton M.D., Bojarski M.T., Dewhirst F.E., Smith M.B. (2004). Structures and biological activity of phosphorylated dihydroceramides of porphyromonas gingivalis. J. Lipid Res..

[bib0040] O-Brien-Simpson N., Veith P.D., Dashper S.G., Reynolds E.C. (2003). Porphyromonas gingivalis gingipains: the molecular teeth of a microbial vampire. Curr. Protein Pept. Sci..

[bib0041] Okamoto K., Nakayama K., Kadowaki T., Abe N., Ratnayake D.B., Yamamoto K. (1998). Involvement of a lysine-specific cysteine proteinase in hemoglobin adsorption and heme accumulation by porphyromonas gingivalis. J. Biol. Chem..

[bib0042] Orench-Rivera N., Kuehn M.J. (2016). Environmentally controlled bacterial vesicle-mediated export. Cell Microbiol..

[bib0043] Peng X., Cheng L., You Y., Tang C., Ren B., Li Y., Xu X., Zhou X. (2022). Oral microbiota in human systematic diseases. Int. J. Oral Sci..

[bib0044] Perez-Chaparro P.J., Gracieux P., Lafaurie G.I., Donnio P.Y., Bonnaure-Mallet M. (2008). Genotypic characterization of porphyromonas gingivalis isolated from subgingival plaque and blood sample in positive bacteremia subjects with periodontitis. J. Clin. Periodontol..

[bib0045] Rocha F.G., Ottenberg G., Eure Z.G., Davey M.E., Gibson F.C. (2021). Sphingolipid-containing outer membrane vesicles serve as a delivery vehicle to limit macrophage immune response to porphyromonas gingivalis. Infect. Immun..

[bib0046] Sakanaka A., Kuboniwa M., Shimma S., Alghamdi S.A., Mayumi S., Lamont R.J., Fukusaki E., Amano A. (2022). Fusobacterium nucleatum metabolically integrates commensals and pathogens in oral biofilms. mSystems.

[bib0047] Sakanaka A., Kuboniwa M., Takeuchi H., Hashino E., Amano A. (2015). Arginine-ornithine antiporter arcd controls arginine metabolism and interspecies biofilm development of streptococcus gordonii. J. Biol. Chem..

[bib0048] Shibata S., Shoji M., Okada K., Matsunami H., Matthews M.M., Imada K., Nakayama K., Wolf M. (2020). Structure of polymerized type v pilin reveals assembly mechanism involving protease-mediated strand exchange. Nat. Microbiol..

[bib0049] Shoji M., Naito M., Yukitake H., Sato K., Sakai E., Ohara N., Nakayama K. (2004). The major structural components of two cell surface filaments of porphyromonas gingivalis are matured through lipoprotein precursors. Mol. Microbiol..

[bib0050] Shoji M., Sato K., Yukitake H., Naito M., Nakayama K. (2014). Involvement of the wbp pathway in the biosynthesis of porphyromonas gingivalis lipopolysaccharide with anionic polysaccharide. Sci. Rep..

[bib0051] Simon R., Priefer U., Pühler A. (1983). A broad host range mobilization system for *in vivo* genetic engineering: transposon mutagenesis in gram negative bacteria. Bio/Technology.

[bib0052] Simpson W., Olczak T., Genco C.A. (2004). Lysine-specific gingipain k and heme/hemoglobin receptor hmur are involved in heme utilization in porphyromonas gingivalis. Acta Biochim. Pol..

[bib0053] Smalley J.W., Birss A.J., McKee A.S., Marsh P.D. (1991). Haemin-restriction influences haemin-binding, haemagglutination and protease activity of cells and extracellular membrane vesicles of porphyromonas gingivalis w50. FEMS Microbiol. Lett..

[bib0054] Stanton B.A. (2021). Extracellular vesicles and host-pathogen interactions: a review of inter-kingdom signaling by small noncoding rna. Genes.

[bib0055] Teufel F., Almagro Armenteros J.J., Johansen A.R., Gislason M.H., Pihl S.I., Tsirigos K.D., Winther O., Brunak S., von Heijne G., Nielsen H. (2022). Signalp 6.0 predicts all five types of signal peptides using protein language models. Nat. Biotechnol..

[bib0056] Toyofuku M., Nomura N., Eberl L. (2019). Types and origins of bacterial membrane vesicles. Nat. Rev. Microbiol..

[bib0057] Toyofuku M., Schild S., Kaparakis-Liaskos M., Eberl L. (2023). Composition and functions of bacterial membrane vesicles. Nat. Rev. Microbiol..

[bib0058] Veith P.D., Chen Y.Y., Gorasia D.G., Chen D., Glew M.D., O'Brien-Simpson N.M., Cecil J.D., Holden J.A., Reynolds E.C. (2014). Porphyromonas gingivalis outer membrane vesicles exclusively contain outer membrane and periplasmic proteins and carry a cargo enriched with virulence factors. J. Proteome Res..

[bib0059] Veith P.D., Glew M.D., Gorasia D.G., Reynolds E.C. (2017). Type ix secretion: the generation of bacterial cell surface coatings involved in virulence, gliding motility and the degradation of complex biopolymers. Mol. Microbiol..

[bib0060] Veith P.D., Shoji M., Scott N.E., Reynolds E.C. (2022). Characterization of the o-glycoproteome of porphyromonas gingivalis. Microbiol. Spectr..

[bib0061] Vermilyea D.M., Moradali M.F., Kim H.M., Davey M.E. (2021). Ppad activity promotes outer membrane vesicle biogenesis and surface translocation by porphyromonas gingivalis. J. Bacteriol..

[bib0062] Vermilyea D.M., Ottenberg G.K., Davey M.E. (2019). Citrullination mediated by ppad constrains biofilm formation in p. Gingivalis strain 381. NPJ Biofilms. Microbiomes.

[bib0063] Wilson M.M., Bernstein H.D. (2016). Surface-exposed lipoproteins: an emerging secretion phenomenon in gram-negative bacteria. Trends Microbiol..

[bib0064] Xu Q., Shoji M., Shibata S., Naito M., Sato K., Elsliger M.A., Grant J.C., Axelrod H.L., Chiu H.J., Farr C.L., Jaroszewski L., Knuth M.W., Deacon A.M., Godzik A., Lesley S.A., Curtis M.A., Nakayama K., Wilson I.A. (2016). A distinct type of pilus from the human microbiome. Cell.

