# Supplementary material for: An outer membrane vesicle specific lipoprotein promotes Porphyromonas gingivalis aggregation on red blood cells
Source: Curr Res Microb Sci. 2024 Jun 8;7:100249. doi: 10.1016/j.crmicr.2024.100249 (PMC11225709; doi:10.1016/j.crmicr.2024.100249)
Supplement: Supplementary file 1 [file mmc1.pdf]

SUPPLEMENTARY INFORMATION

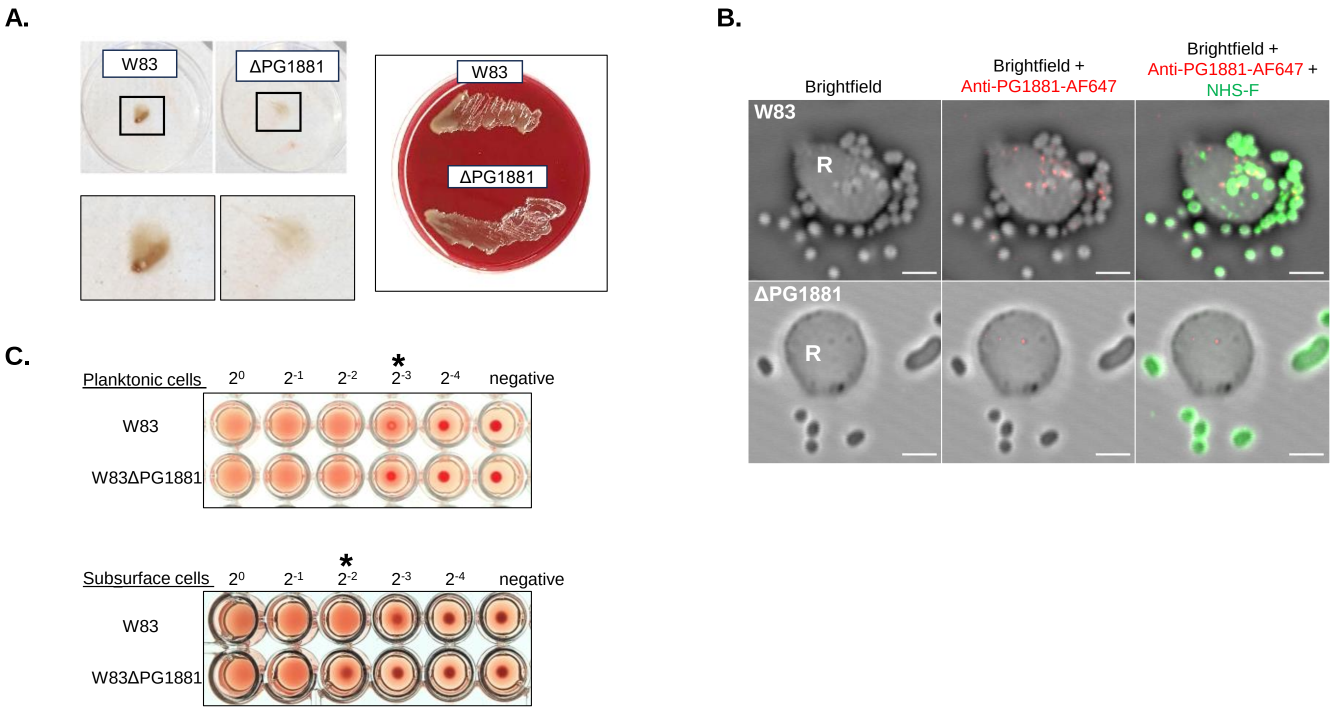

Supplementary Figure 1. PG1881 promotes attachment on RBCs. (A) The left image is representative of parent strain W83 and PG1881 deletion mutant cells grown on the subsurface of soft blood agar plates at 72 hrs, with the black boxes representing zoomed in images. After the removal of the soft agar, W83 cells remaining on the bottom of the plastic had darker pigmentation than the PG1881 deletion mutant, indicating heme accumulation. The right image is representative of W83 and PG1881 deletion mutant grown on the surface of blood agar plates where no differences in pigmentation were observed. (B) Top panels are of W83 during sub-surface growth conditions at 72 hrs using immunofluorescent staining of PG1881 (red fluorescence; top middle and right panels). Bottom panels are of the PG1881 deletion mutant as a control. NHS-Fluorescein was used to stain protein (green fluorescence; right panels). Magnification is 63X with Zeiss Airyscan image processing. R indicates RBC. Scale bar represents 2 µm. (C) Hemagglutination assay on planktonic cells from W83 and PG1881 deletion

mutant show similar activity. Cells grown at the subsurface show hemagglutination activity is higher in W83 compared to the PG1881 deletion mutant.

W83

(4/10) (8/12) (7/10) (13/13) (10/14)

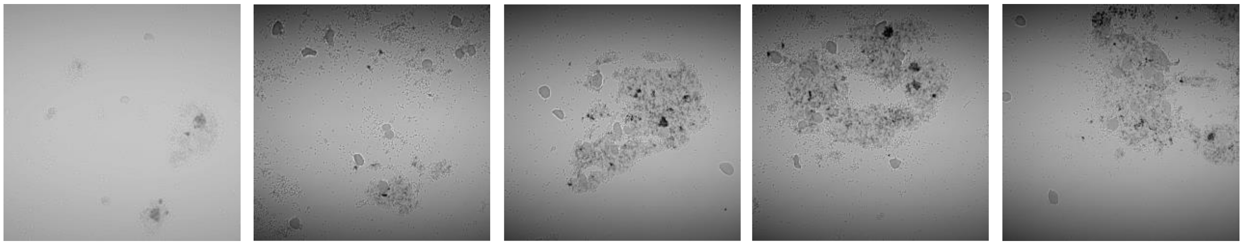

(29/31) (2/5) (2/4) (7/11) (5/6)

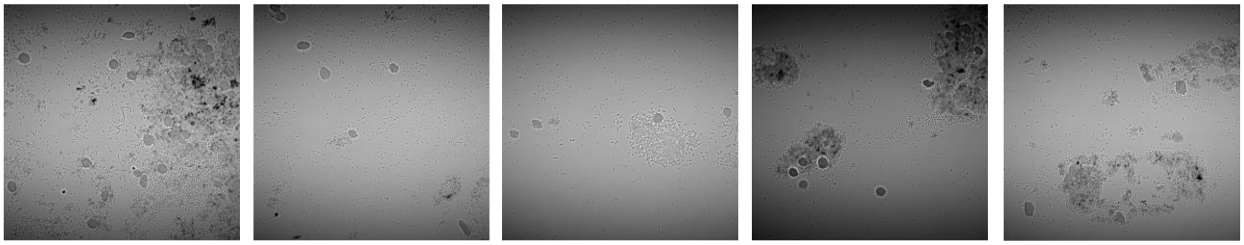

W83ΔPG1881

(2/17) (0/6) (5/11) (0/8) (0/8)

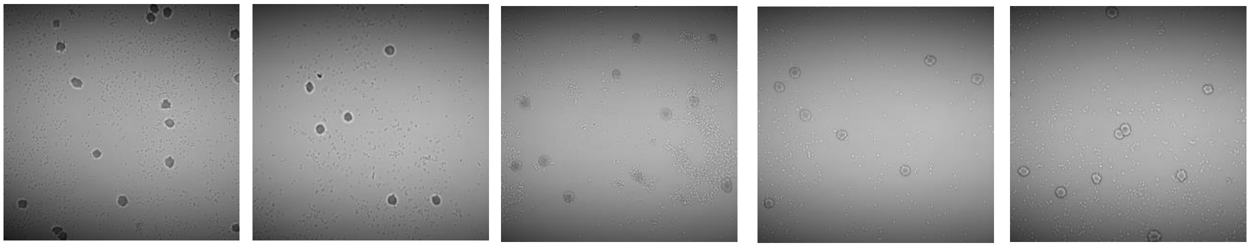

(0/14) (0/12) (0/13) (0/14) (0/11)

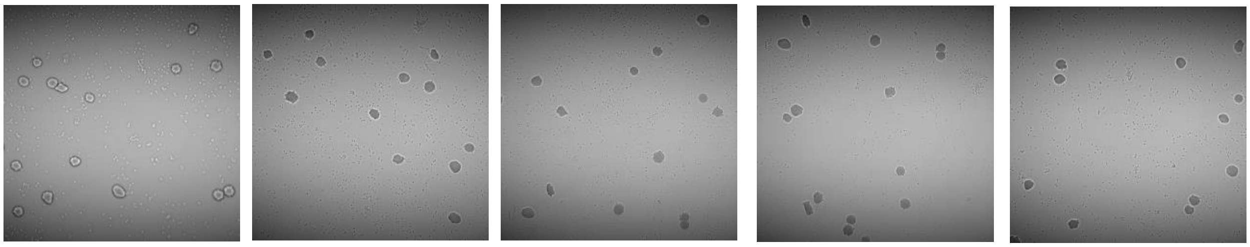

Supplementary Figure 2. PG1881 promotes aggregation of *P. gingivalis* on RBCs. Confocal images of parent strain W83 and W83ΔPG1881 cells grown on the subsurface of soft blood agar plates at 72 hrs used for quantification. The number of red blood cells surrounded by more than ten *P. gingivalis* cells (first digit) was divided by the total number of red blood cells (second digit) and expressed as a percentage.

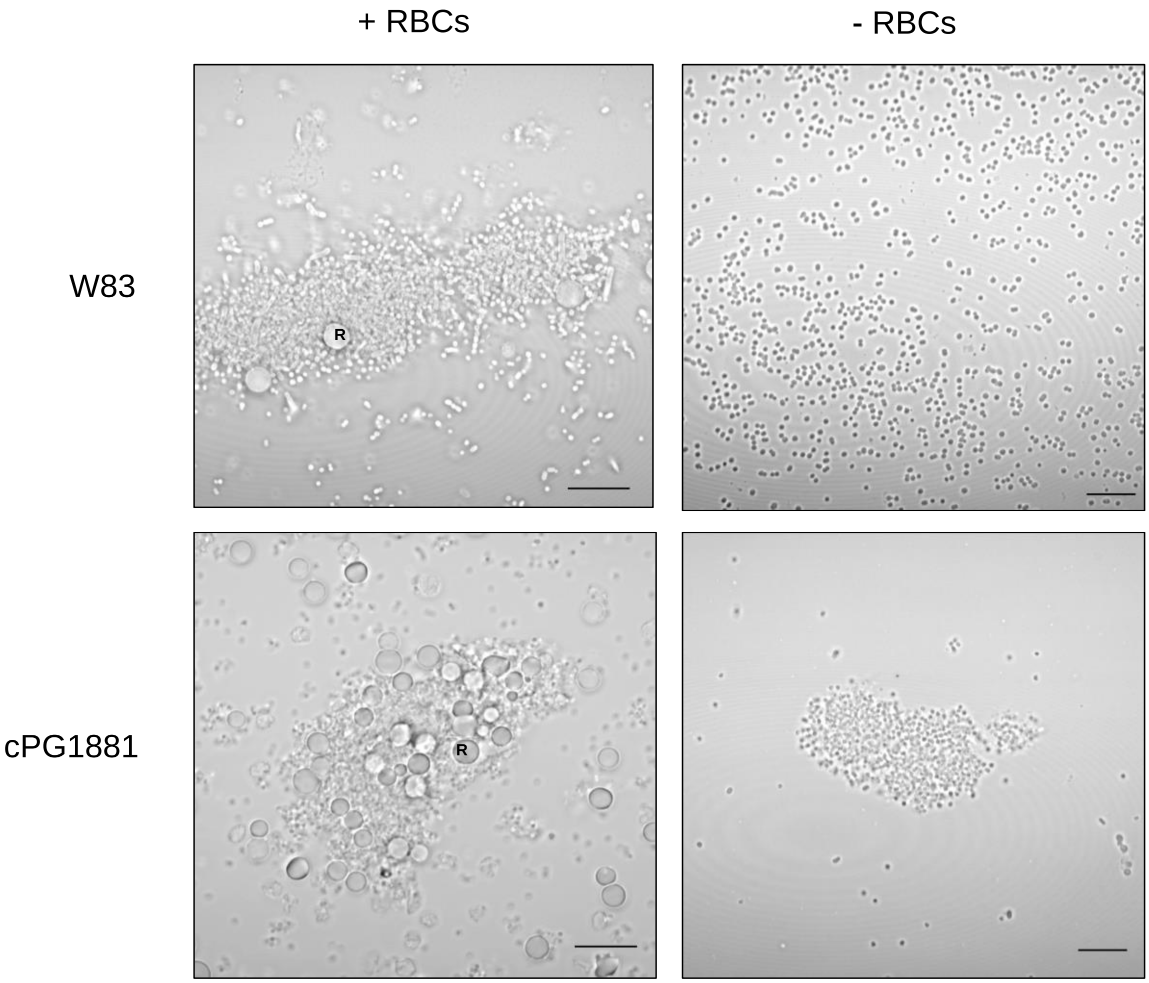

Supplementary Figure 3. The presence of RBCs and PG1881 promotes aggregation of *P. gingivalis*. Top panels: representative confocal images of parent strain W83 grown at the subsurface of soft agar in the presence or absence of RBCs for 72 hrs. Bottom panels: representative confocal images of the complemented strain, in which PG1881 is expressed from a plasmid under the control of a low-level constitutive promoter *groES*, grown in the same conditions described above. For all images, magnification is 63X. R indicates RBC. Scale bar represents 10 µm.

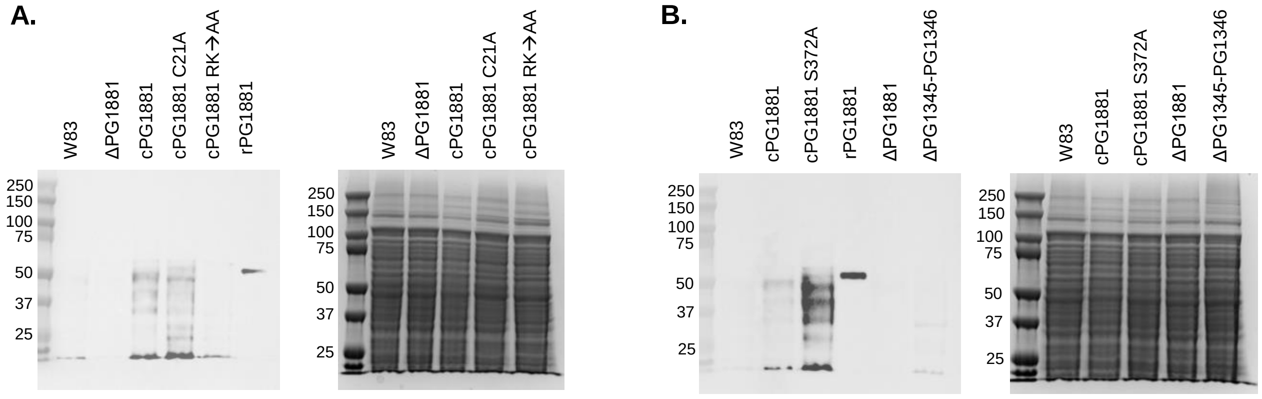

Supplementary Figure 4. PG1881 is an OMV-specific lipoprotein. (A) Western blot analyses of whole cell lysates using PG1881 antibody from parent strain W83, W83ΔPG1881, complemented PG1881 (cPG1881), and site-directed mutagenesis strains potentially involved in lipidation (C21A) or proteolytic processing (RK →AA) of PG1881. On the right is the corresponding Coomassie stain gel. (B) Western blot analyses of whole cell lysates using PG1881 antibody from strains involved in O-glycosylation of PG1881 (S372A and ΔPG1345-PG1346). On the right is the corresponding Coomassie stain gel.

A.

MLTKLKTLLLGCSLACIGFSCSNHPVLTNADDVEQPLD
SGYITLDLRSNLHLSRKGGTHDPLQSVRRITFLFFHET
DSKLLLSRTVEPTSDLSFDLKIPKQNYRLAVLVNSGSS
YAAIIPEILLPTTAIQATSQTLFESFAAYETGNITSESEHS
VTMANDQGLIKLLSTQIVDKKSQLSEASRLSVNVEPCL
ARVLVVGKPTISGGEYTGDVSCYVIDVVPQRIYPLRHL
AKLSSGTNEAYGDNSPLADRYASSWAEESIAAGVAYN
NVYGYVKADMFDNPVAATKMQEKKTDFNLNQVAIYTK
ESTVNPKNYFTAYVPRVVLRAKYVPHGIPGVKPDEGW
IEFQGRKMSLEQFKKYVDNPVSAGMALAD**S**IKKAKAD
NSLVYTGGFVSHGIQFYYKSQNYYAIPIRHFDDEKAPN
KDSYGRFGLVRNNEYILSVKSITGAGSPIVPPVSTTEAI
EKEGYLPASIAVNQTTAHEQDVDL

B.

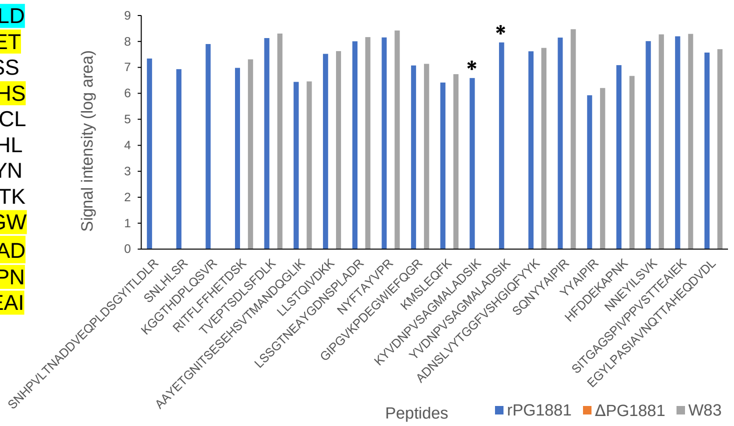

Supplementary Figure 5. PG1881 post-translational modifications include proteolytic processing and O-glycosylation. (A) Mass spectrometry coverage of PG1881. Only peptide masses without predicted post-translational modification were selected for analysis. Regions highlighted in yellow indicate the peptide masses found in both rPG1881 and *P. gingivalis* W83 OMV samples. Regions highlighted in blue were found in rPG1881 sample but missing in *P. gingivalis* W83 OMV sample. Bold S indicates O-glycosylated residue. (B) Signal intensity of the peptide masses in the samples of rPG1881, W83 OMV and ΔPG1881 OMV. The peptide masses were not detectable in ΔPG1881 OMV sample. The peptides that could be O-glycosylated in *P. gingivalis* were marked by an asterisk (*).
